# Gallbladder Cancer With Jaundice: Surgery Versus No Surgery

**DOI:** 10.7759/cureus.30594

**Published:** 2022-10-22

**Authors:** Sugumaran K, Vasistha M Jajal, Phani K Nekarakanti, Devendra Choudhary, Hirdaya H Nag

**Affiliations:** 1 Surgical Gastroenterology, Govind Ballabh Pant Institute of Postgraduate Medical Education and Research, New Delhi, IND

**Keywords:** liver resection, survival, surgery, jaundice, advanced gallbladder cancer

## Abstract

Background

Patients with gallbladder carcinoma (GBC) and jaundice have a poor prognosis. The surgical management of these patients is controversial. There is a dearth of studies comparing curative surgical resection (CR) versus non-curative resection/palliation (NCR) in patients with GBC and jaundice. Hence, this study aimed to compare the outcomes between CR and NCR in these patients.

Methodology

This was a retrospective study on patients with GBC and jaundice managed by a single surgical unit at a tertiary care center in northern India from May 2009 to March 2021. These patients were grouped into CR or NCR. The clinical demographical profile and overall survival (OS) were compared between the groups.

Results

A total of 82 patients with GBC and jaundice were managed during the study period. The final study cohort included 59 patients (CR, n = 34; NCR, n = 25) after excluding patients with metastatic disease (n = 23). Common bile duct infiltration was seen in 61.7% and 84% of CR and NCR patients, respectively (p = 0.062). The overall tumor-node-metastasis staging between the two groups was similar (p = 0.296). The median OS of CR was significantly better in CR than NCR (20 months vs. six months; p = 0.001). The median OS was better in CR than NCR patients who received systemic chemotherapy (22 vs. 12 months; p = 0.001) or did not receive chemotherapy (14 months vs. three months; p = 0.001).

Conclusions

Patients with GBC and jaundice have better significant survival after CR than NCR alone.

## Introduction

Gallbladder cancer (GBC) patients usually present at an advanced stage owing to their ill-defined symptoms [1]. Jaundice in GBC patients is one obvious sign of advanced disease, and around 30-60% of patients with GBC can have this symptom at presentation with a resectability rate of around 7-49% [2-4]. The presence of jaundice has been considered a sign of inoperability in many studies [5-9]. Initially, D'Angelica et al. reported a median survival of 19 months in patients with GBC with jaundice undergoing extensive resection [1]. Subsequent studies from different parts of the world have demonstrated improved survival with resection in properly selected patients [2-4,9]. Surgical resection appears to be the only curative option; however, its role is not clearly defined in this set of patients. Although obvious, patients who undergo curative resection might have a better prognosis, but without clinical evidence, this remains a speculation. Most studies in the literature have compared the survival of GBC patients with and without jaundice. There are no studies signifying the surgical resection and survival outcome in advanced GBC with jaundice who were operated on with curative intent compared to those managed with non-surgical/palliative interventions. Thus, we aimed to study and compare the surgical and survival outcomes of advanced GBC patients with jaundice who underwent curative resection and those managed without curative resection/palliative care.

## Materials and methods

This was a retrospective study done using a prospectively maintained database of a single unit at a tertiary care center. A total of 174 patients diagnosed with stage III and stage IV GBC (as per AJCC 8th edition) were treated for 12 years from May 2009 to December 2021.

The study included patients with GBC and clinical symptoms and signs of biliary obstruction, i.e., elevated bilirubin levels and elevated alkaline phosphatase levels. Patients with metastatic disease (M1, n = 23), preoperatively or intraoperatively diagnosed, were excluded. Patients without any evidence of biliary obstruction were excluded from the study (n = 92).

**Figure 1:**
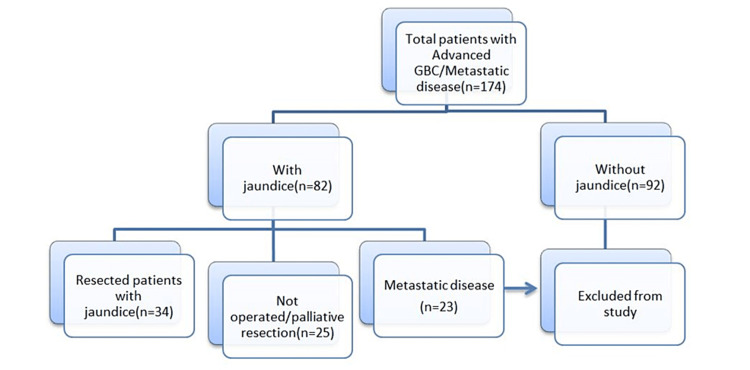
Flow diagram of patient selection. GBC: gallbladder cancer

The final study population was divided into two groups, i.e., the curative resection group and the non-curative resection/palliation group (Figure 1).

The following data were collected for all patients from our prospectively maintained records: gender, age, performance status, comorbidities, clinical features, carcinoembryonic antigen (CEA) and carbohydrate antigen 19-9 (CA 19-9) levels, primary tumor characteristics (location, T category, nodal status, locoregional involvement, distant metastasis, histological grade according to the eighth edition of the AJCC staging manual), details of chemotherapy, biliary drainage, and surgery-associated variables (type of procedure, duration, blood loss, postoperative complications). Complications were graded based on the Clavien-Dindo classification [10]. The baseline clinical characteristics, demographic parameters, cause of jaundice, pathological characteristics, resectability, use of systemic chemotherapy, and overall survival were analyzed and compared.

Outcomes

The primary outcome of the study was overall survival, defined as the date of diagnosis of GBC to the date of death event due to any cause. The secondary outcomes were morbidity, mortality, R1 resection rates, and the effect of adjuvant chemotherapy on overall survival.

Patient management

Patients with suspicion of GBC were evaluated and staged with contrast-enhanced computed tomography (CECT) of the abdomen and pelvis. In the presence of obstructive jaundice, elevated alkaline phosphatase levels, and common bile duct (CBD) involvement on CECT, a magnetic resonance cholangiopancreatography (MRCP) was performed. Preoperative biliary drainage was done for patients with bilirubin levels >3 mg/dL, cholangitis, expected delayed in a waiting period, or planned for chemotherapy. Biliary drainage was done either endoscopically or using the percutaneous method. Patients underwent definitive surgery two to three weeks after preoperative biliary drainage. The benign causes of obstructive jaundice in patients with GBC, such as stones, were cleared endoscopically before resection. In the presence of M1 disease, fine-needle aspiration cytology (FNAC) was obtained to confirm it. Endo-ultrasonography (EUS)-guided FNAC of the inter-aortocaval (IAC) and para-aortic nodes were performed, present if any. Patients were given palliative care in the presence of metastases in these regions (M1) and were excluded from the study. All patients deemed with a resectable disease underwent staging laparoscopy initially and proceeded for either curative or non-curative resection.

Curative Resection Group

All those diagnosed with GBC with jaundice underwent curative resection.

Non-curative Resection/Palliation Group

This group includes patients with GBC and jaundice and in whom definitive curative resection was not possible due to main portal involvement, contralateral vessels involvement, poor performance status, or patients who were unwilling for major surgery. These patients underwent palliative procedures such as gastrojejunostomy, colo-colic bypass, or segment 3 bypass, depending on the predominant symptoms without curative resection.

Surgical procedure

The standard procedure was en-bloc cholecystectomy with segment 4b, 5 liver resection (anatomic bi-segmentectomy or extended liver wedge resection of 2-3 cm) and lymph node dissection along with stations 8, 9, 12a, 12 b, 12 c, and 12 p [11]. A frozen section for the cystic duct margin was sent, and CBD excision was done if positive. CBD excision was also routinely done in all cases if there was macroscopic evidence of infiltration and extensive nodal disease with CBD compression. Those with vascular or extensive liver infiltration were treated with extended right hepatectomy/major liver resections. Further, patients with obvious infiltration of adjacent organs underwent multi-visceral resections such as duodenal sleeve/duodenectomy, colonic sleeve/segmental colonic resections, distal gastrectomy, and hepatopancreaticoduodenectomy.

Follow-up

Patients were followed-up every three months with a physical examination, blood CEA and CA 19-9 levels, and abdominal ultrasonography during the first year, followed by every six months thereafter. Palliative chemotherapy was given if they underwent non-curative resection or adjuvant chemotherapy in those with curative resection after the medical oncologist, surgeons, and patient’s informed consent. CECT abdomen was done yearly. Recurrences were identified either clinically or using radiological imaging. If patients could not turn up to our routine outpatient department (OPD) follow-up, their status was traced telephonically.

Statistical analysis

Data analysis was done using SPSS version 28.0 (IBM Corp., Armonk, NY, USA). Parametric numerical data were represented as mean ± standard deviation and compared with Student’s t-test. The non-parametric numerical data were expressed as median (interquartile range) and compared with a Mann-Whitney (U) test. The categorical and ordinal data are represented as percentages and compared using the Chi-square or Fisher’s test. Survival analysis was done using the Kaplan-Meier method and log-rank test to compare the significance. A p-value <0.05 (two-tailed) was considered statistically significant. Survival curves were constructed using MedCalc software (version 20, Ostend Belgium).

## Results

A total of 59 patients with advanced GBC and jaundice were included in the final analysis after excluding the patients meeting the exclusion criteria. Of them, 34 patients had a curative resection, and 25 patients had either non-curative resection or palliation.

The curative resection group had a significantly younger age group (p = 0.043), good performance status patients (p = 0.001), and lower CA 19.9 levels (p = 0.008) than the palliation group. The presenting complaints, causes of jaundice in patients with GBC, and symptom duration were similar between the groups. Endoscopic drainage was the most common biliary drainage method in the resected group, whereas percutaneous transhepatic biliary drainage (PTBD) was most commonly used in the unresected group (Table 1).

**Table 1:** Baseline clinical characteristics. GBC: gallbladder cancer; TLC: total leucocyte count; ALP: alkaline phosphatase; CEA: carcinoma embryonic antigen; CA 19-9: carbohydrate antigen 19-9; CD: Clavien-Dindo; EUS: endoscopic ultrasonography; FNAC: fine-needle aspiration cytology; AST: aspartate aminotransferase; ALT: alanine aminotransferase; ECOG: Eastern Cooperative Oncology Group; IGBC: incidental gallbladder cancer; CDC: choledochal cyst; PTBD: percutaneous transhepatic biliary drainage *: values expressed in median (interquartile range); **: expressed in n (%), #: p-value significant.

Variables	GBC with jaundice		P-value
	Resected (n = 34)	Unresected (n = 25)	
Age*	49 (28–63)	53.7 (35–66)	0.043^#^
Gender**			
Male	10 (29.4)	11 (44)	0.247
Female	24 (70.6)	14 (56)	
ECOG**			
0	1 (2.9)	1 (4)	0.001^#^
1	11 (32.4)	2 (8)	
2	19 (55.9)	7 (28)	
3	3 (8.8)	14 (56)	
4	-	1 (4)	
Presenting complaint**			
Pain	22 (64.7)	14 (56)	0.498
Jaundice	7 (20.6)	6 (24)	0.755
Loss of weight and appetite	5 (14.7)	5 (20)	0.592
IGBC with jaundice**	6 (17.6)	0	0.106
Symptom duration*	1–12 (3.5 months)	0.1–12 (3.1 months)	0.489
Cause of jaundice**			
CBD infiltration	21 (61.7)	21 (84)	0.348
LN mass-encasing CBD	1 (2.9)	1 (4)	
Mirrizi	8 (23.5)	2 (8)	
CBD stone	2 (5.9)	1 (4)	
Distal CBD growth	1 (2.9)	-	
CDC	1 (2.9)	-	
EUS FNAC **	1 (2.9)	9 (36)	0.001^#^
Stenting**	15 (44.1)	21 (84)	0.002^#^
Endo stenting	12 (35.3)	5 (20)	0.2
PTBD	3 (8.8)	16 (64)	0.000^#^
Neoadjuvant therapy**	4 (11.8)	-	0.007^#^
Adjuvant therapy**			
No	12 (35.3)	12 (48)	0.326
Yes	22 (64.7)	13 (52)	
Operative time(minutes)*	338 (150–600)	75 (30–180)	0.000^#^
Blood loss (mL)*	300 (100–550)	55 (5–300)	0.000^#^
Biochemical parameters*			
Haemoglobin, g/dL	10.8 (7–17.9)	10.1 (7.6–13.4)	0.495
TLC, cells/m^3^	10.4 (3.1–22.4)	8 (3.7–12.7)	0.013^#^
Bilirubin, mg/dL	3.8 (0.2–24.8)	6.05 (0.2–14)	0.059
ALP, IU/L	359 (29–2,035)	313 (92–839)	0.854
CA-19-9, IU/mL	153 (3–2,227)	188 (3.4–1,452)	0.008^#^
CEA, ng/mL	3.7 (0.6–12)	8.3 (1.2–68)	0.078
Platelets, ×10^5 ^cell/mL	2.52 (1.19–4.65)	2.26 (1.12–3.6)	0.304
PT INR	1.10 (0.9–1.33)	0.9 (0.8–1.2)	0.009^#^
AST, IU/L	108.0 (22–897)	65.9 (16–168)	0.307
ALT, IU/L	131.0 (14–1,162)	56.8 (13–180)	0.623
Morbidity and mortality**			
CD1	10 (29.4)	4 (16)	0.246
CD2	15 (44.1)	3 (12)	
CD 3a	3 (8.8)	-	
CD 5	6 (17.6)	-	

The patients in the curative resection group had a lower T stage than the palliation group (p < 0.001). However, the overall TNM stage was similar between the groups (p = 0.296). The resectability rate in the study cohort was 41.5%. Patients who received chemotherapy were comparable between the two groups. The R1 resection rate was around 14.7%, and most of them had a positive liver margin. The recurrence rate was around 55%, with hilar recurrence being the most common (Table 2).

**Table 2:** Pathological data and outcome. GBC: gallbladder cancer; CBD: common bile duct; AJCC: American Joint Committee on Cancer *: values expressed in median (interquartile range); **: expressed in n (%); #: p-value significant.

Variables	GBC with jaundice		P-value
	Resected (n = 34)	Unresected (n = 25)	
Tumor type**			
Adenocarcinoma	30 (88.2)	25 (100)	0.076
Adenosquamous	3 (8.8)	-	
Adenocarcinoma with neuroendocrine differentiation	1 (2.9)	-	
Tumor site**			
Fundus	7 (20.5)	2 (8)	0.385
Body	3 (8.8)	2 (8)	
Neck	5 (14.7)	9 (36)	
Fundus and body	4 (11.8)	4 (16)	
Body and neck	10 (29.4)	6 (24)	
Fundus body and neck	5 (14.7)	2 (8)	
Positive margin**			
R0 resection-	29 (85.2)	-	
R1 resection-	5 (14.7)	-	
T stage AJCC8**			
T2b	3 (8.8)	-	
T3	26 (76.4)	7 (28)	0.000^#^
T4	5 (14.7)	18 (72)	
Stage AJCC8**			
Stage III	14 (41.1)	7 (28)	0.296
Stage IV	20 (58.9)	18 (72)	
CBD involvement**	25 (73.5)	23 (92)	0.144
Vascular involvement**	3 (8.8)	23 (92)	0.000^#^
Duodenal/Stomach**	12 (35.29)	17 (68)	0.013^#^
Pancreas**	1 (2.9)	2 (8)	0.384
Colon**	6 (17.6)	6 (24)	0.549
Number of adjuvant cycles*	0–6 (2.2)	0–6 (1.3)	0.163
Recurrence**			
Present	19 (55.9)	-	
Not known	6 (17.6)	-	
Survival analysis*			
Overall survival (median)	20 months	6 months	0.001^#^
With adjuvant chemotherapy	22 months	6 months	0.000^#^
Without chemotherapy	16 months	3 months	0.001^#^

The most common surgical intervention performed in the resected group was extended cholecystectomy with segment IV b and segment V resection with CBD excision (Table 3).

**Table 3:** Surgical interventions performed. ECW: extended Cholecystectomy-wedge resection; CBD: common bile duct; CDC: choledochal cyst

Resected group		Unresected group	
Type of procedure	n (%)	Type of procedure	n (%)
Extended cholecystectomy S4b + S5 resection	1 (2.9)	Palliative gastric/colonic bypass	4 (16)
ECW	7 (20.6)	Palliative biliary bypass	1 (4)
Extended cholecystectomy S4b + S5 resection with CBD excision	10 (29.4)	Trial dissection (attempted curative resection)	2 (8)
ECW with CBD excision	7 (20.6)		
Mesohepatectomy with CBD excision	1 (2.9)		
Mesohepatectomy with distal gastrectomy and CBD excision	1 (2.9)		
Hepatopancreaticoduodenectomy	2 (5.9)		
Extended right hepatectomy with CBD excision	2 (5.9)		
ECW with CBD, gastric, and colon resection (multivisceral)	2 (5.9)		
ECW with CDC excision	1 (2.9)		

**Figure 2:**
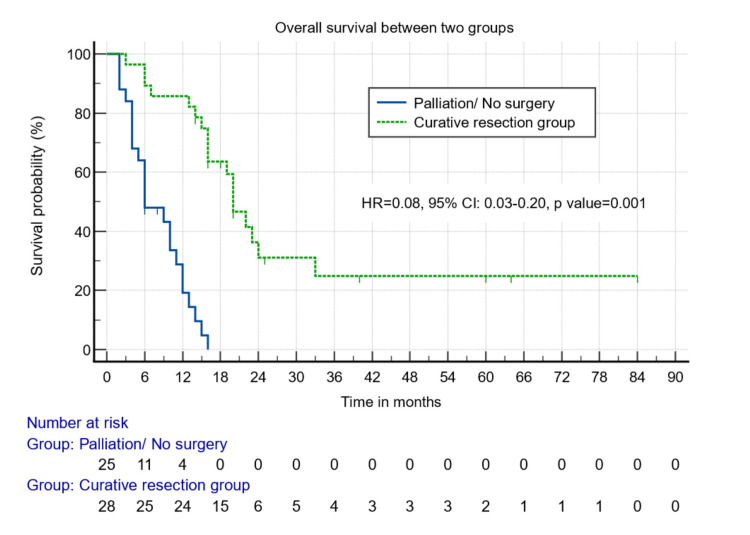
Survival analysis between those who underwent surgical and non-surgical/palliative resection.

The postoperative mortality rate in the curative resection group was around 17.6% (n = 6), and most of these patients underwent major liver resections and HPD. The median overall survival was 20 months in the curative resection group vs. six months in the palliation group (p = 0.001) (Figure 2).

**Figure 3:**
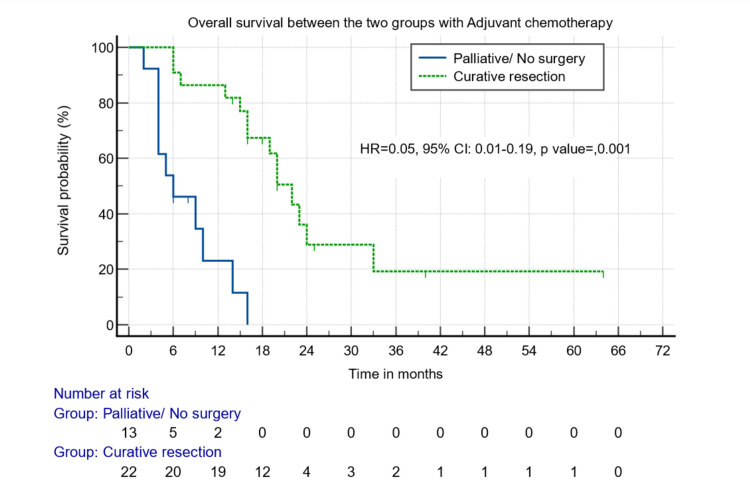
Survival analysis between those who underwent surgical and non-surgical/palliative resection and received adjuvant chemotherapy.

**Figure 4:**
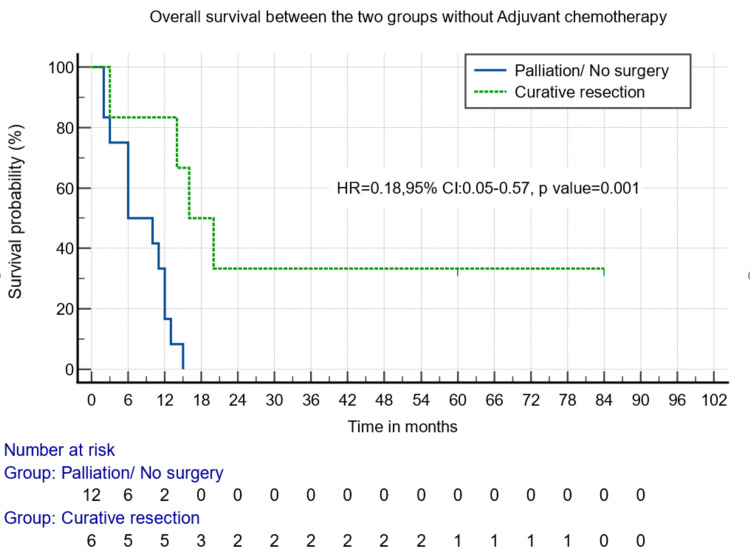
Survival analysis between those who underwent surgical and non-surgical/palliative resection and did not receive adjuvant chemotherapy.

The curative resection group had better median overall survival than the palliative group when the patients received either chemotherapy (22 months vs. six months, p = 0.000) (Figure 3) or not (16 months vs. three months, p = 0.001) (Figure 4).

## Discussion

The presence of jaundice in GBC patients usually indicates advanced disease, and the role of surgery in these patients is controversial. Studies by Chen et al. and Kang et al. show the feasibility of surgical management in advanced stage IV GBC patients [12,13]. However, there are no studies in the literature studying the survival and outcomes of advanced GBC patients with jaundice. Most studies in the literature have compared survival outcomes in patients with and without jaundice undergoing resection [14-16].

The management of advanced GBC with jaundice has been poorly defined in literature due to the aggressive nature and low curative resection rates of the disease. The usually reported incidence of curative resection in GBC with jaundice is around 7-30% [16]. Tran et al. reported a resectability rate of 30.6% [15]. Yang et al. showed a 76.6% resectability rate, which is higher than other studies; however, this study had only 47 patients with jaundice [9]. Another study by Goel et al. reported a resectability rate of 62% using neoadjuvant chemotherapy. Moreover, 44% of these patients had benign etiology as the cause of obstructive jaundice [17]. The resectability rate in our study was around 41.5%, even in advanced cases and without the use of neoadjuvant chemotherapy in most cases. The perioperative mortality rate was 17.6% in our study, and all these patients underwent major liver resections and hepatopancreaticoduodenectomy. Goel et al. reported a mortality rate of only 4.2% with the use of neoadjuvant chemotherapy in 50% of their patients [17]. However, no major liver resections were performed in their study, probably due to downstaging after neoadjuvant chemotherapy. Miyazaki et al. also showed a high mortality rate of 27% when extended right hepatectomy was performed in patients with hilar involvement, mainly due to postoperative hepatic failure [18]. Thus, in this group of patients with jaundice, major liver resections may increase mortality and should be avoided.

Most patients in our study in the resected group belonged to stage III and stage IV disease (AJCC eighth edition). Almost half of the patients (47.1%) in the surgical group had stage IVB disease (AJCC eighth edition) in our study, mainly due to N2 disease. This shows that surgical resection is still feasible even in these advanced stages. In GBC with jaundice patients, the R1 resection rate was found to be consistently higher from the initial studies by Miyazaki et al. [18] in 1996 of 70% to recent studies by Tran et al. [15] in 2017 (48% R1 resection) and Chaudry et al. [19] in 2021 (61% R1 resection). However, studies by Nishio et al. and Nasu et al. (2016) reported R1 resection rates of 16% and 11%, respectively [4,14]. The R1 resection rate in our study was around 14.7%. Hence, despite having a higher chance of R1 resection, most of these patients were able to achieve lower rates of R1 resection. Most of these cases had positive liver margins after resection.

The recurrence rate of GBC jaundice patients is usually high, as seen in the study by Goel et al. of 53% [17]. The recurrence rate in our study was around 56%, and most of them had hilar recurrence, followed by liver lesions. The median survival of GBC in jaundiced patients is 10-14 months [3,9,19]. However, the median survival was 20 months in the resected group and six months in the unresectable group in our study.

Even though most of our patients in the resected group had stage III and stage IV disease, our study has shown that curative resection may increase survival. Adding to this, patients who received adjuvant chemotherapy in the resected group had a median survival of 22 months, and those in the unresected group had a median survival of six months. While those who did not receive adjuvant chemotherapy had a significantly lower median overall survival of 16 months in the resected group and three months in the unresected group. In the study by Goel et al. [17], median overall survival of 32 months was seen using both neoadjuvant and adjuvant chemotherapy though many patients had benign etiology for jaundice.

There are only a few studies in the literature studying the surgical management and outcomes in GBC with jaundice. However, many of these studies have not addressed the impact of chemotherapy on these subsets of patients. Though obvious that patients in the jaundice group have advanced disease and resection improves survival if resectable, there is no evidence available in the literature. No studies compare the survival outcomes in jaundiced GBC patients treated with curative intent and non-surgical/palliative intervention. Until now, only one study has studied the impact of neoadjuvant chemotherapy in GBC jaundice patients [17]. Thus, further randomized control trials are needed to study the role of perioperative chemotherapy in these patients. Our study has compared the survival outcomes in resectable and unresectable groups and has also shown the effect of adjuvant chemotherapy on survival in these two groups. Our study has a few limitations, low sample size, the retrospective nature, and its own inherent bias. The chemotherapy regimen and protocol were not standardized between the groups in our study.

## Conclusions

GBC patients with jaundice usually have advanced disease with low resectability rates, high R1 resection rates, and morbidity with major liver resections. Those who underwent surgical resection with curative intent had a survival benefit compared to those with unresected disease/palliative intervention. Moreover, in the unresectable/palliative group, patients who received chemotherapy had improved survival. Thus, with multimodality management in patients of GBC with jaundice, survival can be improved.

## References

[1] D'Angelica M, Dalal KM, DeMatteo RP, Fong Y, Blumgart LH, Jarnagin WR (2009). Analysis of the extent of resection for adenocarcinoma of the gallbladder. Ann Surg Oncol.

[2] Agarwal AK, Mandal S, Singh S, Bhojwani R, Sakhuja P, Uppal R (2007). Biliary obstruction in gall bladder cancer is not sine qua non of inoperability. Ann Surg Oncol.

[3] Regimbeau JM, Fuks D, Bachellier P (2011). Prognostic value of jaundice in patients with gallbladder cancer by the AFC-GBC-2009 study group. Eur J Surg Oncol.

[4] Nishio H, Ebata T, Yokoyama Y, Igami T, Sugawara G, Nagino M (2011). Gallbladder cancer involving the extrahepatic bile duct is worthy of resection. Ann Surg.

[5] Piehler JM, Crichlow RW (1977). Primary carcinoma of the gallbladder. Arch Surg.

[6] Hawkins WG, DeMatteo RP, Jarnagin WR, Ben-Porat L, Blumgart LH, Fong Y (2004). Jaundice predicts advanced disease and early mortality in patients with gallbladder cancer. Ann Surg Oncol.

[7] Patkar S, Ostwal V, Ramaswamy A (2018). Emerging role of multimodality treatment in gall bladder cancer: outcomes following 510 consecutive resections in a tertiary referral center. J Surg Oncol.

[8] Kapoor VK (2006). Gallbladder cancer: a global perspective. J Surg Oncol.

[9] Yang XW, Yuan JM, Chen JY (2014). The prognostic importance of jaundice in surgical resection with curative intent for gallbladder cancer. BMC Cancer.

[10] Dindo D, Demartines N, Clavien PA (2004). Classification of surgical complications: a new proposal with evaluation in a cohort of 6336 patients and results of a survey. Ann Surg.

[11] Acharya MR, Patkar S, Parray A, Goel M (2019). Management of gallbladder cancer in India. Chin Clin Oncol.

[12] Chen C, Wang L, Zhang R (2019). Who benefits from R0 resection? A single-center analysis of patients with stage Ⅳ gallbladder cancer. Chronic Dis Transl Med.

[13] Kang MJ, Song Y, Jang JY, Han IW, Kim SW (2012). Role of radical surgery in patients with stage IV gallbladder cancer. HPB (Oxford).

[14] Nasu Y, Hirano S, Tsuchikawa T, Shichinohe T (2016). Aggressive surgery for locally advanced gallbladder cancer with obstructive jaundice: result of a prospective study. Dig Surg.

[15] Tran TB, Norton JA, Ethun CG (2017). Gallbladder cancer presenting with jaundice: uniformly fatal or still potentially curable?. J Gastrointest Surg.

[16] Mishra PK, Saluja SS, Prithiviraj N, Varshney V, Goel N, Patil N (2017). Predictors of curative resection and long term survival of gallbladder cancer - a retrospective analysis. Am J Surg.

[17] Goel M, Gupta AM, Patkar S (2021). Towards standardization of management of gallbladder carcinoma with obstructive jaundice: analysis of 113 cases over 10 years at a single institution. J Surg Oncol.

[18] Miyazaki M, Itoh H, Ambiru S (1996). Radical surgery for advanced gallbladder carcinoma. Br J Surg.

[19] Chaudhary RK, Higuchi R, Yazawa T (2021). Resectional surgery in gallbladder cancer with jaundice-how to improve the outcome?. Langenbecks Arch Surg.

